# Microbial communities and their role in enhancing hemp fiber quality through field retting

**DOI:** 10.1007/s00253-024-13323-y

**Published:** 2024-11-05

**Authors:** Eliane Bou Orm, Anne Bergeret, Luc Malhautier

**Affiliations:** 1 Polymers, Composites and Hybrids (PCH), IMT Mines Alès, 6 Avenue de Clavières, 30100 Alès, France; 2 Laboratoire des Sciences des Risques (LSR), IMT Mines Alès, 6 Avenue de Clavières, 30100 Alès, France

**Keywords:** Retting, Hemp, Lignocellulosic fibers, Bacterial and fungal communities, Biochemical composition

## Abstract

**Abstract:**

The current development of industrial hemp “*Cannabis Sativa* L.” fibers for technical textiles and industrial applications requires high-quality fibers with homogeneous properties. However, several factors have been reported to influence the fibers’ intrinsic properties, including a post-harvest process known as retting. This process plays a crucial role in facilitating the mechanical extraction of fibers from hemp stems. Retting involves the degradation of the amorphous components surrounding the fiber bundles enabling their decohesion from stems. Microorganisms play a central role in mediating this bioprocess. During retting, they colonize the stems’ surface. Therefore, the biochemical components of plant cell wall, acting as natural binding between fibers, undergo a breakdown through the production of microbial enzymes. Although its critical role, farmers often rely on empirical retting practices, and considering various biotic and abiotic factors, resulting in fibers with heterogenous properties. These factors limit the industrial applications of hemp fibers due to their inconsistent properties. Thus, the purpose of this review is to enhance our comprehension of how retting influences the dynamics of microbial communities and, consequently, the evolution of the biochemical properties of hemp stems throughout this process. Better understanding of retting is crucial for effective process management, leading to high-value fibers.

**Key points:**

*• Retting enables degradation of cell wall components, controlling fiber properties.*

*• Microbial enzymatic activity is crucial for successful decohesion of fiber bundles.*

*• Understanding retting mechanisms is essential for consistent fiber production* ***.***

## Introduction

The current environmental challenges occurring from the excessive use of global resources like petroleum and water, coupled with carbon dioxide emissions, are increasing climate change and raising alarms among environmental concerns (Bourmaud et al. 2018). To address these issues, it appears necessary to develop new bio-based materials by using renewable resources, such as fibrous plants like flax, hemp, jute, and kenaf, within the context of sustainable development and eco-conception (Bourmaud et al. 2018; Kaur and Kander 2023). In fact, compared with synthetic fibers (glass, carbon, and metallic fibers), lignocellulosic fibers derived from fibrous plant have many advantages due to their abundance, biodegradability, availability, and low-cost (Thyavihalli Girijappa et al. 2019). In addition, these fibers have specific mechanical properties (high tensile strength and stiffness) competitive with those of synthetic fibers (Bourmaud et al. 2018; Chabbert et al. 2023). Therefore, plant fibers can be used in the production of textiles and biocomposites for various industrial applications (building materials, automotive industries, etc.) (Thyavihalli Girijappa et al. 2019).

In this context, the hemp plant has recently been attracting increasing interest worldwide due to its sustainable growth and versatile industrial usability (Ahmed et al. 2022; Yano and Fu 2023). Hemp, native to Central Asia, is belonging to the *Cannabaceae* family and the *Cannabis* genus (Hourfane et al. 2023). It is considered one of the oldest domesticated crops in the world used in various civilizations (Yano and Fu 2023). Hemp is eco-friendly and has a fast growth with the ability to produce high biomass output (Visković et al. 2023). The cultivation of hemp is characterized by its drought resistance, as well as its low requirements for fertilizers and pesticides (Visković et al. 2023). Various products are derived from the entire hemp plant (stem, inflorescences, and seeds) and used in various fields (nutritious food, medicine, and cosmetics) (Zimniewska 2022; Yano and Fu 2023). Concerning fibers, new opportunities for hemp fibers have emerged, expanding beyond its traditional applications in the manufacture of rope and paper (Crini et al. 2020; Amaducci et al. 2015). Then, hemp fibers are currently offering an important economic valorization potential due to their various sustainable applications (Zimniewska 2022).

In the plant stem, elementary fibers, located in the phloem, are linked together to form fiber bundles. These fibers are surrounded by parenchymatous cells and are located between the epidermis and the xylem core, parallel to the longitudinal axis of the stem (Ribeiro et al. 2015). Within these bundles, elementary fibers are embedded in a pectic polysaccharide network, playing a crucial role in maintaining cohesion between the fibers (Ribeiro et al. 2015). To facilitate the extraction of hemp fibers, the hemp plants undergo several transformations (such as in-field transformation, i.e., sowing, harvesting, retting, and post-field transformation, i.e., scutching, hackling, and spinning) (Réquilé et al. 2021). As a result, the final properties of the fibers are significantly influenced by all these steps, especially the retting process (Réquilé et al. 2021).

The field retting (or dew-retting) is a key traditional process that occurs after plant harvest (Chabbert et al. 2023). Field retting is considered as the oldest method of retting and is the most used method for fiber extraction in Europe (Akin 2013). This is because it is an inexpensive and easy-to-apply process compared to other alternative retting methods (water, chemical, enzymatic, and physical) (Zimniewska 2022). Field retting process is a key step in fiber processing. It consists of laying the plant stems on the soil and exposing them to local environmental conditions for several weeks (2 to 10) (Mazian et al. 2018; Bou Orm et al. 2024a). This retting period facilitates the progressive dissociation of fiber bundles through the enzymatic action of microorganisms (e.g., fungi and bacteria) that colonize the stems on plant cell wall components (Bleuze et al. 2018). Hydrolytic enzyme activities facilitate the degradation of parenchymatous cells, separating the fiber bundles from the surrounding stem tissues (Djemiel et al. 2020). Then, these enzymes contribute progressively to a reduction in the cohesion between the fibers by degrading the pectin-rich middle lamellae that link the elementary fibers.

The effectiveness of the retting process is highly dependent on climatic conditions and the farmer’s experience (Pisupati et al. 2021). Hence, uncontrolled weather conditions and the farmers that rely on empirical knowledge to manage retting result in fibers with heterogenous properties (chemical and physical) that can affect their mechanical properties (fiber fineness, tensile strength, etc.) (Mazian et al. 2018). The primary drawback of field retting is the absence of reliable methods to assess the retting degree, posing an unacceptable risk for farmers of obtaining inconsistent fiber quality. Farmers assess the stem degree of retting by examining the color qualitatively (uniform dark gray color), by manually manipulating the stems to assess the ease with which the woody core and fibers separate, or by qualifying the feeling of softness of the fibers by hand (stiff or soft or medium, for example) (Horne 2012; Djemiel et al. 2017; Bou Orm 2023). These traditional indicators do not allow precise monitoring of retting. Moreover, scientific studies on retting management are still partial or incomplete (Chabbert et al. 2023). Therefore, the potential of hemp fibers to meet industrial demand, especially in applications demanding high fiber stability and homogeneity, is limited by the insufficient information on controlling the intrinsic properties of the fibers during retting. In addition, it is crucial to note that the properties of the fibers exhibit natural variations within each plant, regardless of the variety or retting method (Amaducci et al. 2015).

Furthermore, poor retting management can impact fiber quality (Mazian et al. 2019). Over-retting can result in the alteration of the mechanical properties of the fibers through the degradation of their crystalline cellulose structure (Mazian et al. 2018). In contrast, insufficient retting (or under-retting) results in poor separation of fiber bundles, making subsequent extraction steps more difficult and impacting both the fiber yield and quality (Akin 2013).

A better knowledge of the main actors of the retting process, which are the microbial communities, is required for better management of this process. Several studies have focused solely on evaluating the effect of retting on the intrinsic properties of fibers (Liu et al. 2015; Placet et al. 2017; Mazian et al. 2018, 2019), with very few investigating the biological and biochemical mechanisms of hemp retting (Djemiel et al. 2017; Chabbert et al. 2020; Law et al. 2020)—even though the biotic component drives the evolution of intrinsic fiber properties during retting. It is therefore necessary to better understand the biological mechanisms of retting.

Given the current challenges, the main objective of this review is to investigate the dynamics of retting process, targeting the evolution of biochemical characteristics (biochemical component) and examining the microbial communities (biotic component) involved throughout the retting stages. A part of the aim of this review is also to unravel the interactions between both components by investigating potential relationships between the microbial dynamics and functional parameters. This better understanding can provide control, diagnosis, and prevision tools for enhancing retting efficiency and managing the process using innovative strategies to obtain high value fibers.

## Influence of retting on biochemical composition of hemp stems

**Fig. 1:**
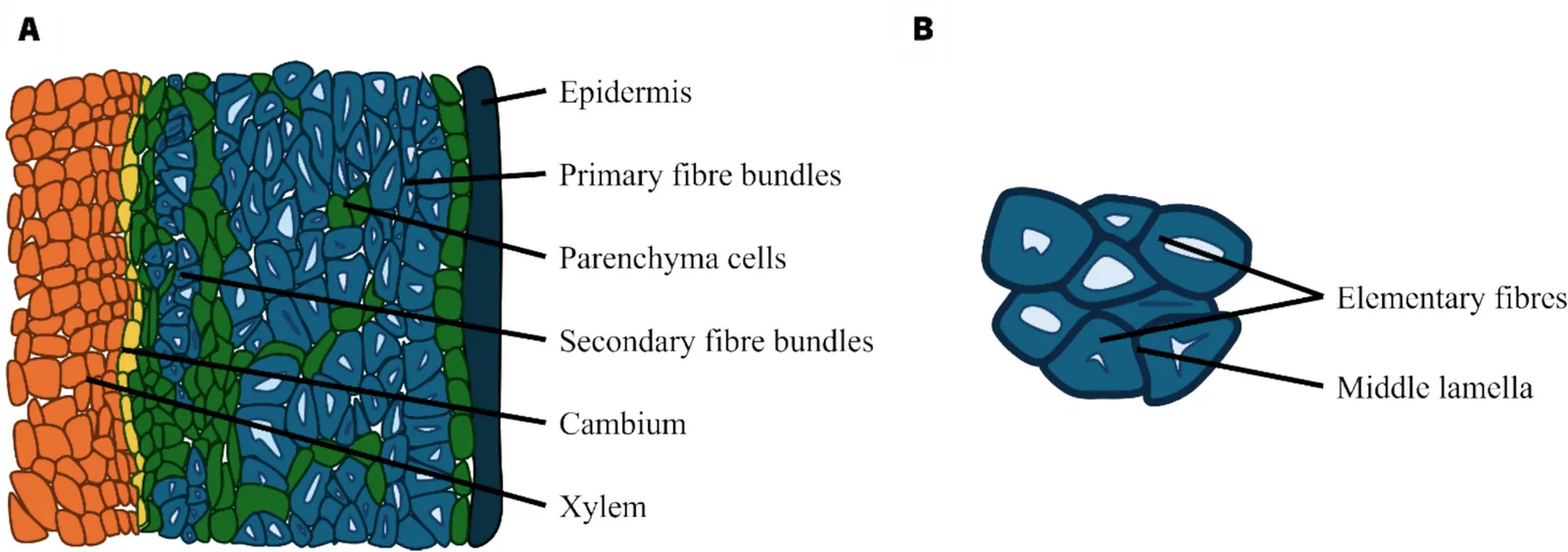
Cross-section of a hemp stem. Organization of the outer tissues (**A**). Middle lamella linking the elementary fibers together (**B**)

Fibers provide strength and stiffness to the plant (Lee et al. 2020). Hemp fibers are cellulosic fibers that are localized under the epidermis, in the phloem, or outer part of the non-woody plant stem and surrounding the core xylem (wood) of the stem (Lee et al. 2020). The xylem and the outer tissues of the stem are divided by a thin layer of undifferentiated cells known as the cambium (Lashermes et al. 2020). Plant fibers are assembled in bundles containing 10 to 40 elementary fibers, with parenchyma cells separating the fiber bundles (Wang et al. 2011). Unlike flax fibers, which consist only of primary fibers, hemp fibers are composed of both primary and secondary fibers (Placet et al. 2014). In general, secondary fibers tend to be thinner, shorter, and possess a higher lignin content compared to primary fibers (Placet et al. 2014) (Fig. 1).

**Fig. 2:**
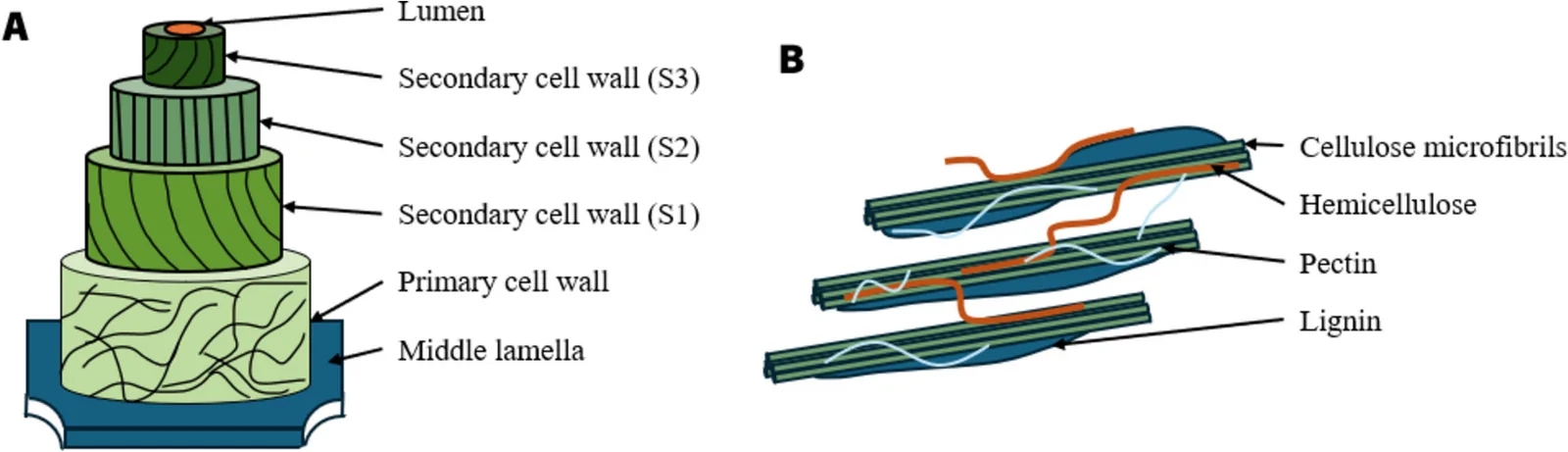
A simplified schematic of the plant cell wall including the three primary layers: the middle lamella, the primary cell wall, and the secondary cell wall (**A**). The main polysaccharides and lignin forming the plant cell wall (**B**) (inspired by Rytioja et al. 2014, with permission)

Most plant fibers consist of cellulose, hemicelluloses, lignin, waxes, and some water-soluble compounds (Zamora-Mendoza et al. 2023). These fibers are created from slender strands of semi-crystalline cellulose enveloped by an amorphous layer of pectin and hemicellulose (Zamora-Mendoza et al. 2023) (Fig. 2). They are composed of a succession of concentric cylinders, featuring a small central channel known as the lumen (Marrot et al. 2013) (Fig. 2). The outer cell wall is known as the primary cell wall representing a thin layer, measuring only 0.1 or 0.2 µm thick. This wall is composed of a network of polysaccharides (90–95%) and proteins (5–10%) (Yang et al. 2016). It is mainly composed of pectins and hemicelluloses but also contains cellulose microfibrils with a dispersed orientation (Sorieul et al. 2016). The secondary cell wall or the inner cell wall (3–13 μm thick) is subdivided into three sublayers (S1, S2 and S3). Each layer is composed of three primary polymer types: cellulose, hemicellulose, and lignin (Placet et al. 2012). The predominant layer, constituting approximately 80% of the total section, is the S2 layer. This layer is characterized by the presence of highly crystalline cellulose fibrils (Marrot et al. 2013). The mechanical properties of fibers result from the presence of this crystalline cellulose and the alignment of the microfibrils to the main axis of the cell (Célino et al. 2014).

Elementary fibers are linked by their middle lamella, an amorphous layer composed primarily of pectic polysaccharides, and could contain hemicellulose, lignin, and a small amount of proteins (Melelli et al. 2020).

The chemical composition of plant fibers was found to be influenced by variations in climate, plant characteristics, and geographical factors (Lee et al. 2020). Furthermore, within the same plant, the biochemical profiles could vary from one fiber to another (Lee et al. 2020). The cell walls of hemp fibers are then rich in cellulose and contain non-cellulosic polysaccharides such as hemicelluloses, pectin, and lignin (Table 1).

**Table 1:** Chemical composition of hemp fibers according to the literature

Hemp variety	Cellulose (%)	Hemicellulose (%)	Pectin (%)	Lignin (%)	References
Fedora 17	75	4	4	2	Crônier et al. (2005)
-	70–92	18–22	1	3–5	Tahir et al. (2011)
Fedora 17	80	12	3	3	Marrot et al. (2013)
-	55–75	4–22	8	2–6	Placet et al. (2017)
Santhica 27	68	16	8	4	Mazian et al. (2019)
-	67–75	16–18	0.8	3–5	Lee et al. (2020)
**-**	57–77	14–22	-	4–13	Zamora-Mendoza et al. (2023)

(-: unspecified hemp variety)

In plant fibers, cellulose which is the most rigid, abundant, and robust organic component plays a primary role in providing stiffness, strength, and stability to the fiber (Yan et al. 2016). It is a homogeneous linear polymer consisting of β-(1 → 4) glycosidic chains aggregated into microfibrils (Synytsya and Novak 2014). These microfibrils stabilized by intra- and intermolecular hydrogen bonds include highly crystalline parts that can resist to both enzymatic and chemical degradation (Synytsya and Novak 2014).

Hemicellulose is the second most abundant component in fibers. Unlike cellulose, hemicelluloses are branched, non-crystalline heteropolysaccharide with β-(1 → 4) linked saccharides and include xylans, xyloglucans, mannans, and glucomannans (Ward 2015). It is an amorphous component, characterized by a non-homogenous structure, and exhibits a high affinity for water due to its hydrophilic nature (Farhat et al. 2016; Berglund et al. 2020). Hemicellulose is strongly linked to cellulose through hydrogen bonds and lignin through covalent bonds (Araujo et al. 2023). Hemicelluloses function as a matrix for cellulose microfibrils; nevertheless, it is highly susceptible to thermal degradation, biodegradation, and moisture absorption (Lee et al. 2020).

Pectins are mainly amorphous polysaccharides composed of polymers of galacturonic acid (Khan et al. 2021). They are found in the primary cell wall and the middle lamella surrounding the fiber bundles (Marrot et al. 2013).

Lignin is a complex polymer characterized by its highly branched amorphous structure. It serves as a binding agent in microfibrils, contributing to the plant’s overall rigidity (Lee et al. 2020). It fills the spaces in the cell wall between the cellulose, hemicellulose, and pectin components, forming covalent bonds with these polysaccharides (Speight 2020). Unlike pectins and hemicelluloses, lignin is highly resistant to microbial degradation (Grgas et al. 2023).

The main goal of the retting process is to remove the fibers’ amorphous components including pectin, hemicellulose, lignin, and waxes, which bind the fibers together in the plant stem. By degrading these components, retting increases the cellulose content of the fibers, thereby enhancing their overall quality.

The degradation of the plant wall components is due primarily to the microorganisms’ activity, bacteria, and fungi to obtain the absorbable forms of nutrients (Bleuze et al. 2018; Djemiel et al. 2020). This degradation could be enhanced under favorable climatic conditions such as high humidity and precipitation levels and optimal temperatures around 20 °C which favors the development of microbial communities at the stem surface (Jankauskiene et al. 2015; Robador et al. 2016).

The pectic elements, which are the main “cementing” components between fibers, are the first components targeted by the microorganism activities during retting (Bou Orm et al. 2024a). Biochemical investigation by using adequate methods (American Society for Testing and Materials (ASTM) standards) provides reliable data (relative abundance values) about the temporal evolution of these complex polysaccharides (Mazian et al. 2018; Bou Orm et al. 2024a). The pectin content gradually decreases during retting, especially at the first weeks of this process (Mazian et al. 2019; Bou Orm et al. 2024a). The content of hemicelluloses also decreases during retting but to a lesser extent compared to pectins (Mazian et al. 2019; Réquilé et al. 2021; Bou Orm et al. 2024a). This is attributed to the structural complexity of hemicelluloses, making them less susceptible to microbial degradation than pectins. Lignin remains relatively stable during retting. However, some studies (Liu et al. 2017; Placet et al. 2017; Mazian et al. 2019) have shown that the content of lignin increases during retting. This can be explained by the fact that lignin is a very diverse polymer, and thus different methods are used to quantify it. During retting, some phenolic or protein components evolved and might be also measured as lignin (Placet et al. 2017). In addition, the decrease in the content of hemicelluloses and pectins could lead to an increase in the relative content of lignin (Placet et al. 2017).

The presence of waxes in the stem epidermal regions serves as barriers, hindering the penetration of microbial enzymes (Akin et al. 2000). Fibers of lower quality showed increased levels of waxes, for example, 1.2% compared to 0.7% in higher quality fibers (Akin et al. 2000; Morrison et al. 2000). Effective retting leads to the removal of surface waxes (Morrison et al. 2000).

After the break down of the amorphous component, an increase in both cellulose content and its crystallinity is observed in retted fibers, resulting in improved mechanical properties, including tensile strength, fiber stiffness, and Young’s modulus (Mazian et al. 2018). The degradation of amorphous component releases the fibers from their bundled attachment, thereby facilitating the defibration of the fibers (Lee et al. 2020). Thus, the fibers obtained are less damaged, enhancing fiber accessibility for further processing steps (Marrot et al. 2013).

For example, the study of Mazian et al. (Mazian et al. 2019) on the field retting of Santhica 27 hemp variety, harvested at the end of flowering stage, using the ASTM standards method, revealed an increase in cellulose content from 68.2% at the beginning of retting to 75.3% after 9 weeks of retting. In addition, it has been reported a decrease in the content of hemicelluloses and pectins from 15.9 to 14.9% and 7.5 to 4.4%, respectively. A notable increase in cellulose crystallinity has been highlighted from 58% at the start of retting to 69% at the end of retting.

In addition, Bou Orm et al. (Bou Orm et al. 2024a) examined the field retting of Futura 75 hemp variety, harvested at the end of flowering stage. Using the ASTM standards method, their findings revealed an increase in cellulose content from 68.4% at the beginning of retting to 79.2% after 6 weeks of retting. Conversely, there was a decline in hemicelluloses from 11.1 to 5.4% and pectins from 7.3 to 1.0%. In particular, the cellulose crystallinity index displayed a slight increase from 63.1 to 66.9% after 2 weeks of retting and then stabilizing until the end of retting.

Other studies that were performed by using high-performance liquid chromatography for carbohydrate determination and Klason method for lignin determination revealed similar data. Retting of USO-31 hemp variety harvested at the beginning of flowering (Liu et al. 2015) revealed that the cellulose relative content exhibited an initial increase from approximately 70 to over 80% (g per 100 g of dry matter) after 50 days (≈7 weeks) of field retting. In addition, a slight decrease in pectin content was observed at the beginning of retting, which then remained stable. In contrast, the lignin content exhibited a consistent increase during the different weeks of retting.

The study of Placet et al. (Placet et al. 2017) observed on the field retting of Fedora 17 hemp variety a decrease in cellulose crystallinity from 77.5 to 72.8% after 5 weeks of field retting and a decline in hemicellulose and pectin relative content from 10.9 to 9.8% (HPLC method). In addition, this study, using the Klason method, revealed an increase in lignin content from 4.9 to 6.9%.

Even though several studies demonstrate a similar trend in cell wall component evolution during retting, these changes can be influenced by various factors, including hemp variety, retting duration, and the specific experimental methodology employed.

In summary, the biochemical composition of hemp fibers undergoes a significant dynamic transformation during retting, with a decrease in pectins and hemicelluloses relative content and an increase in cellulose relative content. This process enhances fiber separation, improves mechanical and physical properties, and prepares the fibers for various end-use applications in composites, textiles, and other industries (Jankauskiene et al. 2015; Mazian et al. 2019). For that, it is imperative to improve these characteristics through enhanced management of the retting process. In this context, given that retting is a microbial-mediated process, it is crucial to understand the retting microbial composition.

The microbial communities are omnipresent in all ecosystems on the planet and play key roles in various ecological functions (biogeochemical cycling, soil structure, organic matter renewal, pollution control, etc.) (Cabrol and Malhautier 2011; Djemiel et al. 2022; Nikitin et al. 2022; Hellal et al. 2023). From an ecological perspective, lignocellulosic biomass constitutes a complex ecosystem that involves microbial mechanisms comparable to those of other natural ecosystems (Sethupathy et al. 2021). Advances in molecular biology, facilitated by the recent development of numerous tools, have paved the way for this ecological approach (Nizamani et al. 2023). Novel methods contribute to enhancing the ability to explore ecological concerns such as the biodiversity-ecosystem function relationship, overcoming limitations associated with selective culture and phenotypic identification, and their lack of representativeness (Djemiel et al. 2020; Nizamani et al. 2023). As suggested by Djemiel et al. (Djemiel et al. 2020), the plant system with its associated microbial communities’ characteristics and interactions will be influenced by both biotic (species, cultivar, and stages of plant development) and abiotic (pedoclimatic environment and soil physicochemical composition) components.

Even if some works have been reported (Djemiel et al. 2017; Law et al. 2020; Bou Orm et al. 2023, 2024b, submitted), our understanding of the impact of environmental conditions, plant characteristics, and farmer experience (turning of hemp swaths…) on the microbial cell-wall degrading activities is limited. Hence, it seems worthwhile to provide an update on the work carried out and to analyze how the results obtained make it possible to improve our understanding of hemp retting and other fiber plants.

In the following sections, we will discuss the aim studies employing sequencing techniques to improve our understanding of the microbial communities involved in hemp retting. These works aim to identify the primary microbial groups (bacterial and fungal) present during retting and to elucidate their potential functional roles in plant cell-wall degradation. This approach provides new insights for optimizing hemp retting and potentially other fiber plants, highlighting the complex interactions between retting microbial communities with the plant stem and the retting environment.

## Retting microbial communities

**Fig. 3:**
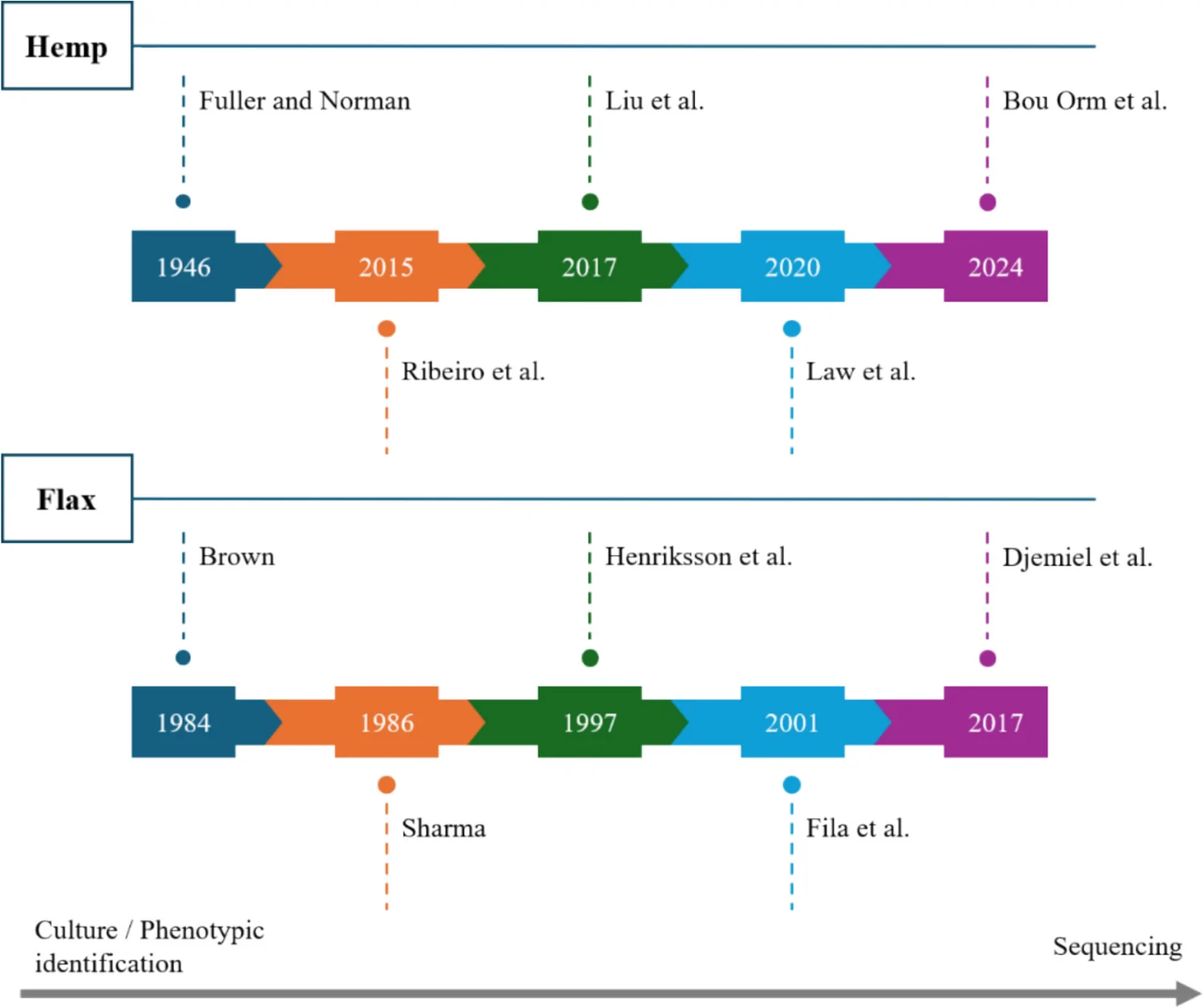
Chronological timeline of the main publications on hemp and flax field retting

In the literature, the microbial communities involved during the hemp field retting process remain poorly studied (Fig. 3). Early investigations, primarily employing culture-based techniques (Fuller et al. 1946), provided initial insights, supplemented by subsequent studies using various methodologies which, while informative, offer only a partial characterization of the complex microbial communities associated with hemp retting (Ribeiro et al. 2015; Law et al. 2020).

Moreover, even though flax has received more attention in terms of research compared to hemp, its microbiome remains relatively under-characterized (Djemiel et al. 2017) (Fig. 3). Despite its agricultural significance and industrial applications, there is still a lack of comprehensive studies elucidating the microbial dynamics of the flax retting (Djemiel et al. 2017; Lashermes et al. 2020; Lee et al. 2020).

Thus, while there is a better understanding of retting process of fiber plants, including both hemp and flax, there remains a significant gap in our knowledge regarding the specific microbial communities driving this process (Bou Orm et al. 2024b, submitted). Closing this gap is essential for optimizing retting methods and improving the overall quality of plant fiber production.

High-throughput sequencing (HTS) technologies have revolutionized the genomics and molecular biology landscape, enabling the swift and accurate sequencing of millions to billions of DNA or RNA molecules (Nizamani et al. 2023). Metabarcoding (or targeted metagenomics) is a specific application of HTS that consists in sequencing an amplicon on a high-throughput platform to characterize microbial diversity from complex/environmental samples (soil, water, and plant material) with high sensitivity and specificity (Djemiel et al. 2020; Nizamani et al. 2023). DNA sequencing, particularly 16S rRNA (bacterial communities), 18S rRNA, and ITS amplicon (fungal communities) sequencing, provides insights into the taxonomic composition of microbial communities by enabling the measure and the evolution of the microbial community structure within an ecosystem (Djemiel et al. 2017, 2020; Law et al. 2020). Thus, the application of targeted metagenomics has enhanced our comprehension of the retting microbiome, enabling the identification of key microorganisms involved during the different stages of retting (Djemiel et al. 2020; Bou Orm et al. 2024b, submitted).

The modifications in fiber chemical composition during field retting are associated with shifts in the relative abundance of the retting microbial community versus time (Liu et al. 2017), highlighting the structural–functional relationships.

## Microbial diversity

### High diversity at the surface of stems

**Fig. 4:**
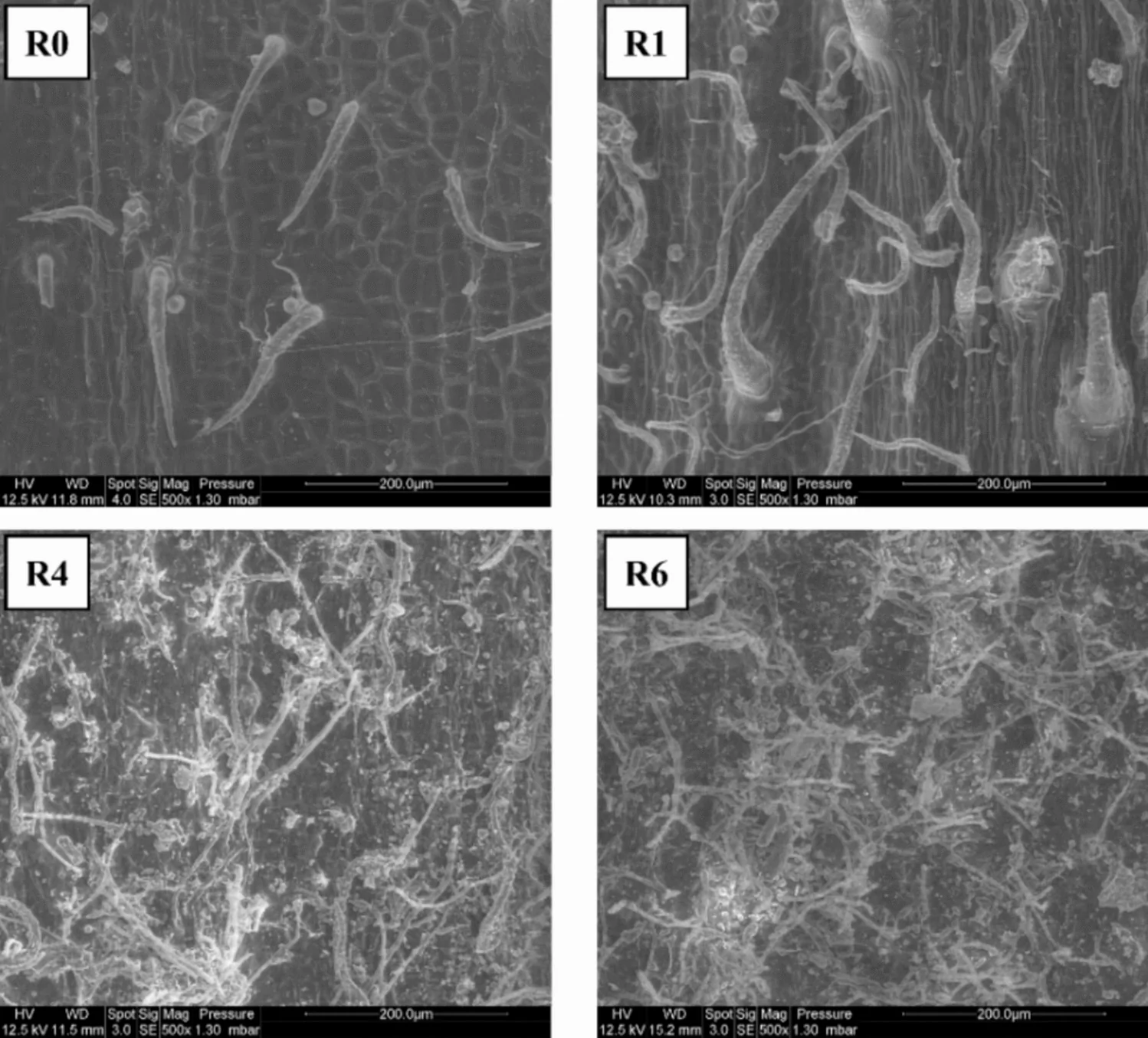
Scanning electron microscopy observations of hemp stem surfaces during hemp field retting stages. R0: unretted stems, R1: stems retted for 1 week, R4: stems retted for 4 weeks, and R6: stems retted for 6 weeks. Scale bar: 200 µm (from Bou Orm 2023)

Microbial colonization during retting involves a gradual proliferation of microorganisms, including both bacteria and fungi, on the surface and within the stem, contributing to the enzymatic degradation of plant components (Fig. 4).

The results of the study of Bou Orm et al. (Bou Orm et al. 2024a) showed that the stem surface before retting (R0) exhibits a few trichomes. However, as retting progresses, microbial communities begin to colonize the stem surface. After 1 week of retting (R1), these microbial communities start to proliferate, and by 4 and 6 weeks of retting (R4–R6), they gradually cover the entire stem surface.

The hemp dew retting communities are usually characterized by high diversity levels. The studies conducted on hemp retting revealed that the microbial communities associated with hemp retting are characterized by a high degree of diversity, with a wide range of taxonomic groups (Law et al. 2020; Bou Orm et al. 2023, 2024b, submitted).

### Relation between diversity and function

It has been largely reported that the diversity of microbial communities, especially the bacterial ones, is impacted by environmental conditions, principally temperature and water content similar to the decomposition of plant litter on a soil surface, which is strongly controlled by mulch moisture (Iqbal et al. 2015; Chabbert et al. 2023). In addition, some findings (Xun et al. 2015) indicate that soil microbial composition and functionality are primarily determined by soil properties rather than microbial inoculum, emphasizing the critical role of environmental factors in shaping these communities. The reverse (i.e., to what extent the diversity drives the macroscopic function in terms of amorphous components biodegradation performance) is still a critical question for ecologists.

This divergence is in keeping with the much-debated discussion on the function of diversity that has occurred in other ecosystems. Indeed, since the key works of Naeem (Naeem et al. 1994) and Tilman and Downing (Tilman and Downing 1994) on different terrestrial ecosystems, the positive effect of diversity on ecosystem function has been evidenced in various environments, at different scales, for widespread or specialized functions (Cabrol and Malhautier 2011). Based on theoretical ecology and modeling, the stabilizing effect of diversity may rely on statistical averaging (known as “portfolio effect”), while the contribution of diversity to improve productivity has been mainly attributed to complementarity between differentiated niches (Tilman and Lehman 2002).

All have trade-off-based mechanisms that allow long-term coexistence of many different competing species (Tilman et al. 2014). These findings indicate the importance of microbial interactions within and among fungal and bacterial communities for enhancing ecosystem performance and demonstrate that the extinction of complex ecological associations below ground can impair ecosystem functioning (Wagg et al. 2019). Given the crucial role microbiomes play in ecosystem functions, results suggest that by limiting the compartmentalization of microbial associations and creating communities dominated by positive associations, increasing stress in the Anthropocene could destabilize microbiomes and undermine their ecosystem services (Hernández et al. 2021).

### Other community factors driving the ecosystem function

In addition to the mentioned factors influencing the retting ecosystem, other community factors play pivotal roles in shaping its functionality. Ecosystem function is closely related to various diversity components, including species richness, evenness, composition, and positive species interactions such as synergism, facilitation, and co-metabolism (Maestre et al. 2012; Orwin et al. 2013; Song et al. 2014; Cabrol and Malhautier 2011). One clear example of ecosystem function during retting is the interaction between pectinolytic microbial species and cellulolytic ones. This synergistic interaction enhances the degradation of lignocellulosic materials, with each microbial group targeting different components of the plant cell wall. While both functional diversity and response diversity are crucial, prioritizing response diversity can enhance the effectiveness of ecosystem management strategies (Ludwig et al. 2018).

Functional redundancy also contributes to the system’s stability (Fetzer et al. 2015; Biggs et al. 2020). It describes the presence of multiple species within the community that perform similar roles (Biggs et al. 2020). For example, during the retting process, if other microorganisms can take over the functions of a lost species, the overall ecosystem’s structure and function could be less affected. Therefore, understanding the degree of functional redundancy within the microbial community is essential for predicting the impact of species loss on the retting ecosystem’s efficiency (Fonseca and Ganade 2001). Several studies on retting observed that when certain species, such as *Basidiomycota species*, were reduced, other microbial species like *Ascomycota species* were able to take over (Djemiel et al. 2020; Bou Orm et al. 2023). This redundancy in the microbial community helped maintain retting efficiency, showing how microbial functions can compensate for the loss of species and support stability in the ecosystem during the retting process.

Productivity outcomes depend on whether the driving force of the ecosystem process leans toward selection or complementarity (Wang et al. 2021). Selection dynamics favor productivity by promoting species that excel in the primary function (Jing et al. 2015; Wang et al. 2021). In such cases, single, highly efficient microorganisms could dominate the process (e.g., pectin-degrading species during retting). On the other hand, complementarity dynamics enhance productivity through diverse species using resources differently or facilitating each other (Jing et al. 2015; Wang et al. 2021). Thus, different retting species with varied capabilities work together, each contributing a specific piece to the degradation of lignocellulosic material, leading to higher productivity.

These nuances highlight the potential multifaceted nature of community dynamics in driving the retting ecosystem’s functionality and emphasize the importance of considering various ecological factors in understanding ecosystem processes.

### Microbial community structure/temporal variability

Complex microbial community structure with bacteria and fungi populations has been revealed, and this paragraph is organized on the results obtained by using molecular approaches. These data were obtained from hemp sampling during dew hemp retting.

Dominant bacterial phyla: *Proteobacteria*, *Bacteroidetes*, *Actinobacteria*, and *Firmicutes* have been identified (Ribeiro et al. 2015; Zhao et al. 2016; Liu et al. 2017; Law et al. 2020; Bou Orm et al. 2023). Large dominance of *Proteobacteria* during retting has been observed.

A more exhaustive work was performed (Bou Orm et al. 2024b, submitted). For retted hemp stems during a 6-week period, 7 bacterial phyla were identified: *Proteobacteria*, *Firmicutes*, *Bdellovibrionota*, *Acidobacteriota*, *Bacteroidota*, *Actinobacteriota*, and *Myxococcota*. *Proteobacteria*, *Bacteroidota*, *Actinobacteriota*, and *Myxococcota* were the 4 major phyla (relative abundance > 1%) with a large dominance of *Proteobacteria* (relative abundance of 93.1% at R0).

A decrease in *Proteobacteria* relative abundance and an increase in *Bacteroidota* relative abundance during the 6 weeks of retting are observed by Bou Orm et al. (Bou Orm et al. 2024b, submitted) and corroborated previous results provided by Liu et al. (Liu et al. 2017) and Law et al. (Law et al. 2020) for hemp retting.

At the class level, the predominant classes during retting are *Alphaproteobacteria* and *Gammaproteobacteria* (Bou Orm et al. 2024b, submitted). Furthermore, it has been demonstrated that the decrease in the relative abundance of the *Proteobacteria* phylum during retting was correlated with a decrease in the relative abundance of the *Gammaproteobacteria* class. Conversely, the increase in the relative abundance of the *Bacteroidetes* phylum during retting was primarily associated with an increase in the relative abundance of the *Bacteroidia* class.

Bou Orm et al. (Bou Orm et al. 2024b, submitted) revealed that during retting, the most dominant bacterial genera were *Pantoae*, *Pseudomonas*, *Sphingomonas*, *Methylobacterium*, and *Rhizobium*, all classified on *Proteobacteria* phylum. Some of them have been reported by Ribeiro et al. (Ribeiro et al. 2015) and Liu et al. (Liu et al. 2017), such as *Pantoa*, *Pseudomonas*, and *Rhizobium*. Other genera have been observed: *Escherichia*, *Rhodobacter*, *Massilia* (Ribeiro et al. 2015; Liu et al. 2017), and *Chryseobacterium* (Law et al. 2020; Liu et al. 2017). According to Liu et al. (Liu et al. 2017), *Massilia aurea* (ß-*Proteobacteria*) was the most dominant species after 7 and 14 days of retting and then decreased after 20 days. The *Pseudomonas* genus (species *argentinensis*, *rhizosphaera*, and *syringae*) decreased after 14 days and until the end of retting. *Shigella sonnei* species (γ-Proteobacteria) decreased after 20 days. In addition, minor but increasing percentages (0 to 3–10% of bacteria) were found for *Erwinia aphidicola* (*Gammaproteobacteria*), *Chryseobacterium scophthalmum* (*Bacteroidota*), *Hymenobacter* species (*Bacteroidota*), and *Pedobacter* species (*Bacteroidota*) (Liu et al. 2017).

The dominance of *Alphaproteobacteria* and *Gammaproteobacteria* has previously been identified during field flax retting (Djemiel et al. 2017; Chabbert et al. 2020). Due to its cellulolytic and pectinolytic activities, *Proteobacteria* can act on the degradation of cellulose and pectin (Liu et al. 2017). *Pseudomonas* genus (*Proteobacteria* phylum) has previously been associated with the retting of fibrous plants (flax, hemp, and jute) (Djemiel et al. 2017; Law et al. 2020; Munshi and Chattoo 2008). *Pseudomonas* species are particularly important in the decomposition of pectin in plant fibers under both aerobic and anaerobic conditions (Zimniewska 2022) and have been also recognized as effective retting agents (Ribeiro et al. 2015). In addition, they can act on the degradation of lignin (Wu et al. 2022) and hemicelluloses (Emami et al. 2002) in plant fibers.

### Fungal retting communities

While bacterial communities play a pivotal role during the retting process, it is equally essential to explore the dynamics of fungal communities during this bioprocess. The protective cuticle layer on hemp stems, composed of lipophilic materials like epoxy-like cutin and cuticular wax, forms a chemically rigid barrier against microbial invasion (Fernando et al. 2019). Fungi play an important role during retting, both by degrading the plant cell walls themselves and by facilitating the entry of other microorganisms such as the bacterial communities, in the cuticle of the plant stems (Law et al. 2020). Fungal hyphae penetrated plant stems using naturally occurring openings in the cuticle layer, such as trichome detachment sites (Bou Orm et al. 2024a). Once inside the stem, hyphae colonized the plant tissue, leading to the mycelial growth (Fernando et al. 2019). In addition, fungal hyphae can penetrate deep into the stem, enhancing the degradation processes of the plant wall components.

Dominant fungal phyla are *Ascomycota* and *Basidiomycota* which have previously been identified during field flax retting (Djemiel et al. 2017; Chabbert et al. 2020). During the first stage of hemp retting (between 0 and 7 days), the fungal community was dominated by *Ascomycota* phylum, which increased significantly after first days of retting (7–14 days). However, the *Basidiomycota* phylum decreased after 1 to 2 weeks of retting (Bou Orm et al. 2024b, submitted; Fernando et al. 2019; Liu et al. 2017; Ribeiro et al. 2015). The most prevalent fungi were primarily associated with the *Cladosporium* (*Ascomycota* phylum) and *Cryptococcus* (*Basidiomycota* phylum) genera. According to the work of Bou Orm et al. (Bou Orm et al. 2024b), the increase in the relative abundance of the *Ascomycota* phylum was mainly related to the increase in the relative abundance of the *Dothideomycetes* and *Sordariomycetes* classes. However, the decrease in the relative abundance of the *Basidiomycota* phylum was mainly due to the decrease in the relative abundance of the *Exobasidiomycetes* class.

Notably, genera such as *Lectera*, *Plectosphaerellaceae_genus* (*Sordariomycetes* class), *Tilletiopsis* (*Exobasidiomycetes* class), *Didymosphaeria*, *Aureobasidium*, *Phoma*, and *Cladosporium* (*Dothideomycetes* class), as well as *Vishniacozyma* and *Filobasidiaceae_genus* (*Tremellomycetes* class), were identified as the most dominant fungal genera during retting. Throughout the different weeks of retting, the genus *Cladosporium* is largely dominant over all other fungal genera. Species of *Penicillium*, *Rhodotorula*, and *Aspergillus* were identified during field retting (Ribeiro et al. 2015). These species are recognized as retting agents. Other fungal genera have been identified by Liu et al. (Liu et al. 2017), such as *Alternaria*, *Cladosporium*, *Gibellulopsis*, *Leptospora*, and *Stemphylium* (*Ascomycota* phylum), as well as *Bulleromyces*, *Cryptococcus*, *Dioszegia*, *Entyloma*, *Rhodotorula*, and *Sporobolomyces* (*Basidiomycota* phylum). *Stemphylium globuliferum* was the most dominant species during retting. In addition, the study revealed increases in percentages for both *Cladosporium uredinicola* and *Alternaria infectoria*. *Cladosporium* species and *Stemphylium* species dominated both the initial and late stages of retting, while *Alternaria* exhibited an increasing trend throughout the entire retting period.

To resume, *Ascomycota* and *Basidiomycota* are the major fungal phyla identified during hemp retting. These phyla present in retted stem samples were associated with hemp and flax field retting (Ribeiro et al. 2015; Djemiel et al. 2017; Chabbert et al. 2020). In addition, these phyla were identified during litter decomposition, as the predominant fungal taxa (Voriskova and Baldrian 2013; Dong et al. 2021). Several studies have demonstrated a shift in the fungal community during retting and plant decomposition, with *Ascomycota* gradually replacing *Basidiomycota* (Voriskova and Baldrian 2013; Djemiel et al. 2017, 2020). This suggests that analyzing the fungal community, particularly the changes in the relative abundance of *Ascomycota* and *Basidiomycota*, could be a valuable bioindicator to track the progress of retting.

### Retting microbial diversity and function

In summary, the retting process of hemp involves complex microbial communities comprising both bacteria and fungi. Bacterial activities were observed to be promoted by fungal mycelia, which involved a variety of phenotypic behaviors associated with nutrient acquisition and transport via fungal highways within the hemp stems (Fernando et al. 2019).

The composition of retting microbial communities undergoes important dynamic changes throughout the retting process. The first stages of retting are the most dynamic, and it is hypothesized that each microbial (bacterial and fungal) population has a distinct role in the sequential degradation of the chemical components of plant stem (Fu et al. 2011). During the first stages of the retting process, fungi contribute to a rapid degradation of the outer layers of the hemp stem. As retting progresses, bacteria accelerate the plant cell wall component’s degradation. The final stages of retting are characterized by a decline in microbial activity as the remaining fiber components become inaccessible to microorganisms (Bou Orm et al. 2024b, submitted). These findings underscore the intricate interplay between bacteria and fungi during the retting process, shedding light on their roles in fiber decohesion and the potential for optimizing industrial hemp retting practices.

Understanding the evolution of the dynamics of microbial communities during retting reveals their potential as bioindicators to track the retting process (Djemiel et al. 2017; Bou Orm et al. 2024b, submitted). The observed shifts in relative abundance across bacterial phyla (*Bacteroidota* vs. *Proteobacteria*) and fungal phyla (*Ascomycota* vs. *Basidiomycota*) offer valuable insights into the retting progress. By monitoring these changes, we can gain early indications of the retting stage, leading to improved retting process control.

### Towards the functional community

#### Retting enzymes

Field retting relies on enzymes secreted by a diverse microbial community present naturally on the soil and on the plant stem (Bou Orm et al. 2023). While this bioprocess has been used for centuries, the specific enzymes involved and their complex interactions remain under investigation. The retting duration corresponds to the period during which microorganisms are actively ensuring a gradual enzymatic degradation of the fiber’s chemical components (Bou Orm et al. 2024b, submitted). These microbial communities possess diverse metabolic capabilities, enabling them to break down various polysaccharides, including pectins, cellulose, hemicellulose, and lignin, the main components of hemp fibers (Chabbert et al. 2020). As an example, fungi belonging to the *Ascomycota* phylum play a key role in retting due to their ability to produce various carbohydrate-degrading enzymes (CAZymes). These enzymes can degrade the plant matter efficiently, making *Ascomycota* fungi highly active contributors during the retting process (Dong et al. 2021). Notably, their pectinolytic activity is crucial for improving fiber decohesion during retting (Gacura et al. 2016; Djemiel et al. 2020). These studies have identified several key genera within this phylum, with *Cladosporium* being the most dominant one during retting. Several *Cladosporium* species are known to produce various lignocellulolytic enzymes. These include cellulases (Srivastava et al. 2020; Moharram et al. 2022), ligninases (Pozdnyakova et al. 2022), xylanases (Ji et al. 2014), and pectinase enzymes (Ribeiro et al. 2015; Moharram et al. 2022). *Basidiomycota* fungi are highly effective in degrading lignocellulosic matter, with a high ability for both cellulose and lignin degradation (Eichlerova et al. 2015; Liu et al. 2017). Thus, understanding the roles of different bacteria and fungi in complex processes like retting requires studying their functional capabilities.

**Fig. 5:**
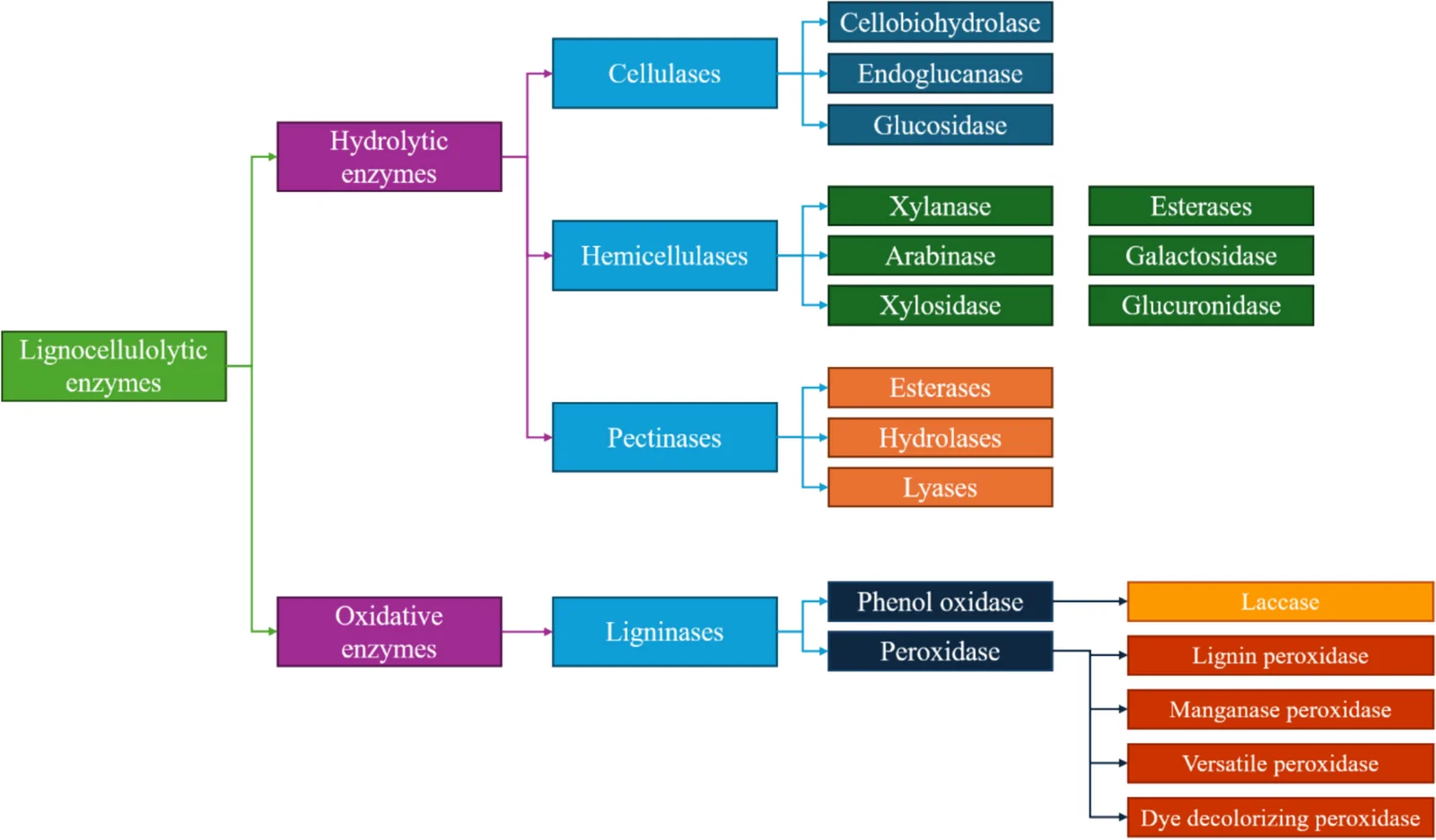
The key enzymes involved in the lignocellulosic degradation process

Two types of enzymatic activities can be distinguished during retting: hydrolytic activities and oxidative activities (Bleuze et al. 2018; Chabbert et al. 2020) (Fig. 5). Hydrolytic enzymes consist of pectinases, cellulases, and hemicellulases, responsible for the degradation of polysaccharides (Chukwuma et al. 2020). Microorganisms also possess a ligninolytic oxidative system (laccases and peroxidases) responsible for the depolymerization of lignin (Kumar and Chandra 2020) (Fig. 5).

The microorganisms present during the initial stages of retting are known for their ability to target available polysaccharides such as pectins and hemicelluloses (Fu et al. 2011). Complex molecules such as cellulose exhibit resistance to degradation, yet cellulolytic fungi and bacteria possess cellulosome enzyme complexes (including endo- and exo-cellulases and β-glucosidases). Thus, in the case of over-retting, these complexes enable them to degrade cellulose (Lakhundi et al. 2015; Bou Orm 2023).

#### Insights into enzyme behavior during retting

The study of Bleuze et al. (Bleuze et al. 2018) investigated the enzymatic activities potentially produced by the retting microbial communities during hemp retting under controlled environmental conditions. This study exhibited that polygalacturonase activity, an enzyme that acts on the pectin degradation, remained high throughout retting, reflecting consistent pectinase activity. Enzymes involved in hemicellulose (α-L-arabinosidase, β-D-galactosidase, and β-D-xylosidase) and cellulose (glucosidase and cellobiohydrolase) degradation showed increased activity primarily in the first 2 weeks, correlating with a decrease in cell wall monomers. However, the increasing cellulolytic activity did not correspond to a reduction in cell wall glucose, suggesting cellulose degradation in parenchyma cells rather than bast fibers. These findings suggest that biological activities primarily target thin-walled cells rich in pectin, hemicellulose, and some cellulose, while cellulose-rich fiber walls remain relatively unaffected.

The study of Chabbert et al. (Chabbert et al. 2020) explored the enzymatic activities potentially produced by the retting microbial communities during flax retting. Enzyme activity measurements were conducted on frozen stems, from which soluble fractions were extracted and used for assays, using various methods including fluorogenic product release and spectrophotometric measurements (Sauvadet et al. 2016; Bleuze et al. 2018). Polygalacturonase exhibited its highest activities immediately after flax uprooting, followed by a gradual decrease throughout the retting process. In addition, the lignin-degrading enzymes, phenol oxidase, and peroxidase exhibited their highest activities at the start of retting and then decreased throughout the retting process. In contrast, other enzymes targeting polysaccharides, such as β-D-galactosidase, β-D-xylosidase, glucosidase, and cellobiohydrolase, showed an initial increase in activity during the first 2 weeks of retting, followed by a decline. The study noted similarities in enzyme dynamics with those observed during controlled hemp retting, where polysaccharide-active enzyme activities peaked in the first 2 weeks before declining (Bleuze et al. 2018). However, the authors cautioned against solely attributing enzyme activities during initial retting stages to microorganisms. The presence of endogenous plant enzyme activity, such as peroxidase enzyme, could influence the observed enzymatic dynamics post-harvest.

More recently, the study of Bou Orm et al. (Bou Orm et al. 2024b, submitted) on field hemp retting revealed distinct enzymatic dynamics, with polygalacturonase exhibiting peak activity during initial retting stages, indicating early pectin degradation, while ß-xylosidase and ß-galactosidase showed increased activity after 4 weeks, suggesting slower hemicellulose degradation. Cellulolytic enzymes peaked towards the end of retting, potentially reflecting late-stage cellulose degradation. In addition, peroxidase and phenoloxidase, associated with lignin degradation, reached their highest levels during the first week of retting. Thus, this study showed a similar trend to flax (Chabbert et al. 2020) and hemp (Bleuze et al. 2018) retting studies, where enzymatic activity increases during retting and stabilizes or decreases as the process progresses. However, in contrast to Bou Orm et al. (Bou Orm et al. 2024b, submitted), these studies did not seem to demonstrate a sequential approach in the production of enzymes. This sequential and time-dependent process involves the degradation of different components of the plant cell wall, with cellulose being the most challenging to degrade due to its crystalline structure, while pectin and hemicellulose are more easily degraded.

A recent study (Ventorino et al. 2024) on the water retting of hemp stems reveals that pre-treatment conditions in the field significantly affect pectinolytic enzymatic activity and alter microbial diversity dynamics during water retting. Hemp stems without pre-treatment showed a marked increase in pectinase activity during retting. Moreover, while initial microbial diversity was high, the retting process selected for specific bacterial populations lead to a more effective pectinolytic microbiota and improved fiber quality. These insights illustrate the crucial role of environmental conditions in optimizing fiber extraction.

The enzymatic activity measurements potentially produced by the retting microbial communities can be complemented by bioinformatic tools, which offer a rapid and comprehensive view of retting microbial functionality. Bioinformatic predictive tools such as PICRUSt offer a powerful solution using the 16S rRNA marker gene sequences (Langille et al. 2013). This approach allows to predict the functional profile of bacterial communities, by identifying the Carbohydrate Active Enzyme (CAZy) families, providing valuable insights into their potential to degrade stem cell wall polymers (Bou Orm et al. 2024b, submitted). Moreover, this tool offers significant advantages over time-consuming measurements of retting enzyme activities by providing a rapid and comprehensive view of microbial functionality (Djemiel et al. 2017; Bou Orm et al. 2024b, submitted).

Djemiel et al. (Djemiel et al. 2017) studied the enzymatic potential during field retting of flax stems by predicting bacterial CAZy families using PICRUSt software. The study identified a variety of enzymes targeting cellulose, hemicelluloses, and pectins. The hydrolytic enzyme potential was higher in the initial stages of retting and decreased in later stages. Despite variations in the stem’s microbiome, the proportion of enzymes targeting different polysaccharides remained constant.

In addition, the study of Bou Orm et al. (Bou Orm et al. 2024b, submitted) emphasizes the use of PICRUSt to predict potential bacterial enzymatic activities during hemp field retting, aiding in understanding microbial functionality. PICRUSt identifies CAZy families from 16S rRNA gene profiles, shedding light on the degradation of stem cell wall polymers. The findings of this study indicate a diverse set of enzymes targeting cellulose, hemicelluloses, and pectins, with distinct trends observed during retting weeks. Notably, cellulolytic enzymes like ß-glucosidase show increasing activity, while others like cellulase fluctuate. Hemicellulolytic and pectinolytic enzymes also exhibit varying activity levels across retting weeks, underscoring the dynamic nature of the retting process. These studies (Djemiel et al. 2017; Bou Orm et al. 2024b, submitted) highlight a gap in predicting fungal enzyme activity, crucial for understanding the complete retting process.

Metagenomics offers a powerful tool to study the composition of microbial communities. Genomics uses DNA-based approaches, such as sequencing gene amplicons encoding rRNA, enabling the determination of microbial community composition and providing insights into its functional potential (Satam et al. 2023). However, this tool lacks insight into which genes are actively expressed (Ferrocino et al. 2023). Thus, while metagenomics is useful for identifying taxonomic composition and providing some insight into functional predictions, its capacity to determine real functional profiles is limited. Therefore, for a more comprehensive understanding of the retting microbiome’s functions, alternative approaches exist. Using other omics technologies such as metatranscriptomics, metaproteomics, and metabolomics allows for the identification of unknown enzymes and provides a better understanding of interactions within the retting functional community (Ferrocino et al. 2023; Djemiel et al. 2022; Bou Orm 2023). Transcriptomics focuses on the study of the transcriptome, the totality of an organism’s RNA. This approach measures gene expression and identifies key metabolic pathways within an ecosystem (Lowe et al. 2017). Proteomics explores the proteome, representing all proteins in an organism, while metabolomics studies the metabolome, examining the entirety of metabolites generated within an organism (Fischer et al. 2013; Clish 2015). Integrating transcriptomics with proteomics and metabolomics serves as a powerful tool to target the active and functional community within an ecosystem (Fischer et al. 2013).

Finally, it is crucial to link biochemical analysis with these biological analyses during retting to establish an accurate understanding of the enzymatic activities produced by microbial communities and their functional implications in the degradation of plant cell wall polymers.

## Concluding statement

Synthetic fibers like carbon and glass are widely used for their performance and lightness, but high costs and environmental issues have spurred interest in natural fibers, which are cheaper and biodegradable (Elfaleh et al. 2023; Thapliyal et al. 2023). While the potential of natural fibers such as hemp fibers has attracted growing interest, challenges remain in achieving widespread commercial success (Zimniewska 2022; Andrew and Dhakal 2022; Kaur and Kander 2023; Mariz et al. 2024). Despite challenges, ongoing research aims to optimize natural fiber properties (Elfaleh et al. 2023; Angulu and Gusovius 2024). Thus, several industries depend on the enhancement of the intrinsic properties of natural fibers to create innovative, environmentally friendly materials with the targeted properties for specific applications (bio-composites, textiles, automotive, construction, etc.) (Müssig et al. 2020; Elfaleh et al. 2023). A critical step in the transformation of hemp fibers is the retting process, which separates the fibers from the stem’s woody core. However, during this process, fibers often exhibit heterogeneous properties due to the influence of various biotic and abiotic factors (Bou Orm et al. 2024b, submitted).

The microbial communities (bacterial and fungal) of retting play a pivotal role in the degradation of non-cellulosic components, liberating the fiber bundles (Bou Orm et al. 2024b, submitted). Effective retting removes non-cellulosic components, enhancing fiber fineness, flexibility, and strength (Sadrmanesh and Chen 2018). However, excessive retting can weaken fibers due to the degradation of cellulose (Feleke et al. 2023). Thus, understanding the microbiological mechanisms of retting is essential for optimizing the process and producing high-quality fibers.

This review has summarized key studies on hemp field retting, focusing on the biochemical evolution of fibers during retting, the microbial communities involved, and their potential enzymatic activities.

Retting influences significantly the biochemical composition of hemp fibers by degrading amorphous components like pectin, hemicellulose, lignin, and waxes, which bind the fibers together, thereby increasing the cellulose content and enhancing fiber quality (Placet et al. 2017; Mazian et al. 2019; Bou Orm et al. 2024a). This degradation process is facilitated by microbial activity, which is influenced by several abiotic and biotic factors. During retting, pectin and hemicelluloses decrease, while cellulose content and its crystallinity increase, improving the mechanical properties of the fibers (Mazian et al. 2019; Bou Orm et al. 2024a).

The microbial communities involved in hemp field retting are poorly understood, with early culture-based studies providing limited insights and more recent methodologies offering only partial characterizations of the complex microbial ecosystems (Bou Orm et al. 2024b, submitted). High-throughput sequencing (HTS) technologies, particularly the metagenomics approaches, have greatly advanced our understanding of retting microbiology, allowing detailed identification of key microorganisms and their potential roles during retting (Djemiel et al. 2017; Law et al. 2020; Bou Orm et al. 2024b, submitted). Bacterial communities during retting are highly diverse, predominantly consisting of *Proteobacteria*, *Bacteroidetes*, *Actinobacteria*, and *Firmicutes*, with significant shifts in their relative abundances during the different stages of retting. Fungal communities, primarily *Ascomycota* and *Basidiomycota*, present also dynamic changes observed throughout the retting stages. These findings suggest that specific microbial populations could serve as bioindicators to optimize retting practices. Furthermore, understanding the interactions between bacteria and fungi during retting is essential for improving hemp fiber production.

Retting enzymes are produced by these diverse microbial communities that degrade plant cell wall components. Two main enzyme activities are identified: hydrolytic (pectinases, cellulases, and hemicellulases) and oxidative (laccases and peroxidases). Studies reveal distinct enzyme activity patterns during retting. Pectic enzymes show high activity throughout, while cellulolytic enzymes peak later, reflecting a sequential degradation process of the plant wall components. Bioinformatic tools like PICRUSt enhance understanding by predicting the functional profiles of the bacterial communities and identifying specific enzymes involved in cell wall polymer degradation. Integrating omics technologies (metagenomics, transcriptomics, proteomics, and metabolomics) provides a comprehensive view of the retting process, linking microbial activity to enzymatic degradation and improving our understanding of fiber transformations.

In summary, it is crucial to control the retting process and determine the optimal retting degree to improve fiber quality and yield sustainably. Developing reliable indicators such as colorimetry can facilitate rapid monitoring of retting dynamics. Understanding the interactions between hemp stems and their associated microbial communities is fundamental. Research on microbial roles in the degradation of plant cell wall components can enhance retting management. Combining statistical methods, predictive modeling, and connected agriculture technologies can lead to the development of real-time monitoring tools. These innovations will equip farmers with a comprehensive retting toolkit to optimize fiber properties and retting outcomes.

## Data Availability

No new data were generated or analyzed in this study. All information discussed is available within the referenced sources.
